# SNORD3A Regulates STING Transcription to Promote Ferroptosis in Acute Kidney Injury

**DOI:** 10.1002/advs.202400305

**Published:** 2024-07-04

**Authors:** Huanhuan Zhu, Junni Wang, Jin Miao, Mingdi Shen, Huijing Wang, Xiaohan Huang, Anqi Ni, Huijuan Wu, Jianghua Chen, Liang Xiao, Shanshan Xie, Weiqiang Lin, Fei Han

**Affiliations:** ^1^ Kidney Disease Center The First Affiliated Hospital, Zhejiang University School of Medicine Institute of Nephrology Zhejiang University Key Laboratory of Kidney Disease Prevention and Control Technology Zhejiang Province; Zhejiang Clinical Research Center of Kidney and Urinary System Disease Hangzhou 310003 China; ^2^ Department of Pathology School of Basic Medical Sciences Fudan University Shanghai 200032 China; ^3^ Children's Hospital, Zhejiang University School of Medicine National Clinical Research Center for Child Health Hangzhou Zhejiang 310052 China; ^4^ The Fourth Affiliated Hospital of School of Medicine and International School of Medicine International Institutes of Medicine Zhejiang University Yiwu 322000 China

**Keywords:** acute kidney injury, ferroptosis, Snord3a, snoRNA, STING

## Abstract

Acute kidney injury (AKI) signifies a sudden and prolonged decline in kidney function characterized by tubular cell death and interstitial inflammation. Small nucleolar RNAs (snoRNAs) play pivotal roles in oxidative stress and inflammation, and may play an important role in the AKI process, which remains elusive. an elevated expression of Snord3a is revealed in renal tubules in response to AKI and demonstrates that Snord3a deficiency alleviates renal injury in AKI mouse models. Notably, the deficiency of Snord3a exhibits a mitigating effect on the stimulator of interferon genes (STING)‐associated ferroptosis phenotypes and the progression of tubular injury. Mechanistically, Snord3a is shown to regulate the STING signaling axis via promoting STING gene transcription; administration of Snord3a antisense oligonucleotides establishes a significant therapeutic advantage in AKI mouse models. Together, the findings elucidate the transcription regulation mechanism of STING and the crucial roles of the Snord3a‐STING axis in ferroptosis during AKI, underscoring Snord3a as a potential prognostic and therapeutic target for AKI.

## Graphical abstract

This study shows the involvement of Snord3a in acute kidney injury (AKI) by promoting STING‐associated ferroptosis. Snord3a, a small nucleolar RNA, exhibits high expression levels in clinical renal biopsies and mouse models of AKI. Mechanistically, Snord3a enhances the STING signaling axis by upregulating the transcription of the STING gene, emphasizing the Snord3a‐STING axis as a promising therapeutic target for AKI intervention.

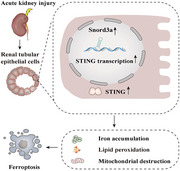

## 1 Introduction

Acute kidney injury (AKI) is a complex syndrome marked by a sudden decline in kidney function, often triggered by factors like ischemia‐reperfusion, nephrotoxins, and sepsis.^[^ ^1^ ^]^ Tubular epithelial cells (TECs), crucial components of the nephron responsible for reabsorption and secretion processes, are highly vulnerable to various stressors due to their unique microvascular environment and elevated metabolic demands. TECs play a determinant role in AKI severity and prognosis as the primary site of injury.^[^ ^2^ ^]^ The initial stage involves TECs injury, followed by regulated cell death (RCD), which may cause kidney injury directly or contribute to modulating inflammation, fibrosis, and the immune response. Modes of regulated cell death, such as ferroptosis, necroptosis, and pyroptosis, may induce impaired regeneration and maladaptive repair, making the critical transition from AKI to chronic kidney disease (CKD).^[^ ^3^ ^]^ Understanding the molecular mechanisms of TECs‐RCD is essential for identifying therapeutic targets in AKI treatment.

Small nucleolar RNAs (SnoRNAs), ranging from 60 to 300 nucleotides, are conserved non‐coding RNAs known for guiding 2′‐O‐ribose methylation and pseudouridylation of ribosomal RNA (rRNAs) and small nuclear RNAs (snRNAs).^[^ ^4^ ^]^ Although renowned for their roles in rRNA modification and ribosome biogenesis, emerging evidence reveals snoRNAs as novel post‐transcriptional regulators influencing rRNA acetylation, tRNA methylation, mRNA abundance, alternative splicing, and translational efficiency.^[^ ^5^ ^]^ While snoRNAs have been implicated in regulating various human diseases, including cancers, genetic disorders, metabolic syndrome, and senescence,^[^ ^6^ ^]^ their roles in renal diseases remain largely unexplored. Recent studies, such as Song et al.’s identification of SNORD63 and SNORD96A as diagnostic biomarkers for renal clear cell carcinoma,^[^ ^7^ ^]^ and Slieker et al.’s report associating circulating snoRNAs with prevalent CKD,^[^ ^8^ ^]^ underscore the crucial link between snoRNAs and kidney functions. However, the biological roles and underlying mechanisms of snoRNAs in AKI remain poorly understood.

Ferroptosis is a regulated form of cell death characterized by iron‐dependent peroxidation of membrane phospholipids, ultimately resulting in membrane destruction.^[^ ^9^ ^]^ Substantial evidence has indicated that ferroptotic cell death in tubular cells emerges as a central feature in the early stage of AKI.^[^ ^10^ ^]^ During this process, entire segments of proximal tubules undergo ferroptosis, leading to heightened lipid peroxidation in the damaged microenvironment.^[^ ^11^ ^]^ Notably, suppressing ferroptotic stress in the proximal tubules also contributes to kidney repair by mitigating inflammation and promoting proximal tubular plasticity.^[^ ^12^ ^]^ However, the specific regulatory mechanisms regulating ferroptosis in the context of AKI remain largely unclear. Indeed, soaring evidence supports the role of non‐coding RNAs in delivering signaling molecules and facilitating intercellular communication to regulate ferroptosis in renal injury. Interestingly, Pan et al. recently identified the ferroptosis‐associated snoRNA SNORA56,^[^ ^13^ ^]^ which stimulates ferroptosis resistance and promotes cell proliferation. Nevertheless, whether and how snoRNA exerts their effects during the AKI process, particularly in the propagation of ferroptosis, remains less clear‐cut.

Here, we highlight the elevated expression of snoRNA Snord3a in TECs, mouse kidneys, and human kidney biopsy samples in response to AKI. We show that Snord3a participates in AKI via promoting STING‐associated ferroptosis. Specifically, Snord3a facilitates the STING signaling axis by promoting the transcription of the *STING* gene. Collectively, our findings underscore the pivotal roles of the Snord3a‐STING axis in ferroptosis during AKI, emphasizing Snord3a as a promising therapeutic target for AKI intervention.

## 2 Results

### 2.1 Snord3a Expression in Response to AKI in TECs, Mouse Kidneys, and Human Kidney Biopsy Samples

**Figure 1:**
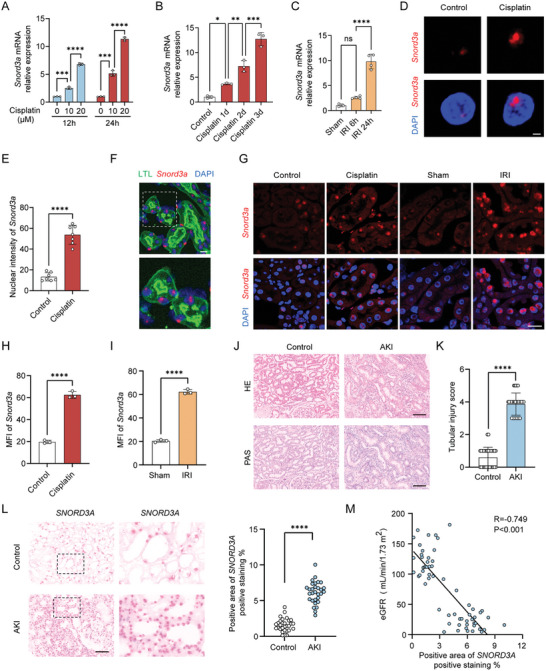
Snord3a expression and characterization in AKI. A) RT‐qPCR analysis of Snord3a expression in the dose and time response of cisplatin‐treated TCMK1 cells (n = 3 for each group). B,C) Mice were intraperitoneally injected with cisplatin (20 mg kg^−1^, n = 3 for each group) or underwent IRI surgery (n = 3 for sham group and n = 4 for IRI group) to induce AKI models. RT‐qPCR analysis of Snord3a expression in the kidney upon cisplatin or IRI models at different time points. D) Representative images of the nuclear localization of Snord3a (red) co‐stained with DAPI (blue) with or without cisplatin (20 µm) for 24 h. Scale bars, 2 µm. E) Quantification of nuclear intensity of Snord3a from D (n =  7 for each group). F) Representative images of the kidney expressing Snord3a (red), and tubules marker (LTL, green) co‐stained with DAPI (blue). Scale bars, 20 µm. The bottom panels are a magnification of hatched boxes. G) Representative images of the kidney in control, cisplatin (3 day), sham, and IRI (24 h) group expressing Snord3a (red) co‐stained with DAPI (blue). Scale bars, 20 µm. H,I) Mean fluorescence intensity (MFI) of Snord3a signals from G (n = 3 for each group). J,K) Representative images of HE and PAS staining of renal sections from healthy donors and AKI patients, and quantification of tubular injury score. Scale bars, 100 µm; n = 30 for each group. L) Representative FISH images and quantification of Snord3a in healthy donors and AKI patients. Scale bars, 100 µm; n = 30 for each group. M) Spearman analysis of the correlation between Snord3a expression and kidney function (eGFR) in the clinical cohort (n = 30 for each group). Data were presented as means ± SD. Statistical analysis was performed using one‐way ANOVA (A, B, and C) or Student's *t*‐test (E, H, I, K, and L). ^*^ *P* < 0.05, ^**^ *P* < 0.01, ^***^ *P* < 0.001, ^****^ *P* < 0.0001.

To investigate the role of snoRNAs in AKI, we conducted snoRNA sequencing on cisplatin‐treated TCMK1 cells, a standard model for inducing injury. The volcano map revealed differential snoRNAs (Figure S1A, Supporting Information), and 9 snoRNAs were identified as significantly upregulated (Log2 Fold Change >2, *P* < 0.05) (Figure S1B; Table S1, Supporting Information). Subsequent analysis of these 9 snoRNAs in cisplatin‐treated TCMK1 and HK2 cells was performed (Figure S1C,D, Supporting Information). qPCR analysis demonstrated that Snord3a exhibited the highest expression in both cisplatin‐treated TCMK1 and HK2 cells (Table S2, Supporting Information). Notably, we observed a time‐dependent and concentration‐dependent increase in Snord3a expression in response to cisplatin‐induced injury in TCMK1 cells (**Figure** **1**A). Moreover, we established cisplatin‐induced and ischemia‐reperfusion injury (IRI)‐induced AKI mice models at different time points to delineate the progressive stages of kidney injury (Figure S2A–F, Supporting Information), and Snord3a was significantly elevated in both models (Figure 1B,C).

Snord3a, a C/D box snoRNA, predominantly localizes to the nucleoli and plays various roles. Therefore, we first explored the subcellular localization of Snord3a in vivo and in vitro. RNA fluorescence in situ hybridization revealed that Snord3a predominantly localized in the nucleus of TCMK1 cells, with a significant elevation in cisplatin‐induced TCMK1 cells (Figure 1D,E). Next, we examined the localization of Snord3a in mouse kidneys, revealing its predominant expression in the nuclei of proximal tubule cells (Figure 1F). Additionally, RNA fluorescence in situ hybridization highlighted a significant elevation of Snord3a levels in both cisplatin and IRI models of AKI (Figure 1G–I).

To comprehensively assess the clinical significance of Snord3a in AKI, we enrolled 30 patients with biopsy‐proven AKI and 30 healthy donors with healthy kidney biopsies. Pathological and clinical characteristics of the enrolled individuals were summarized in Figure 1J,K, and Table S3 (Supporting Information). Remarkably, Snord3a was significantly elevated in the kidney tissues of patients with AKI, and its levels were related to the degree of renal function (Figure 1L,M). Collectively, these findings indicate that Snord3a is upregulated in tubular epithelial cells during AKI, suggesting its involvement in the occurrence and development of AKI.

### 2.2 Snord3a Deficiency Alleviates Renal Injury in AKI Mouse Models

**Figure 2:**
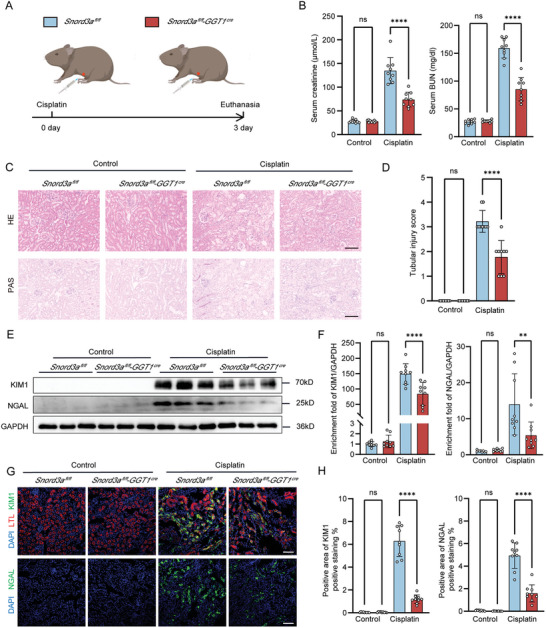
Snord3a deficiency alleviates renal injury in cisplatin‐induced AKI model. A) Schematic representation illustrating the establishment of cisplatin‐induced AKI model in *Snord3a^fl/fl^* and *Snord3a^fl/fl^‐GGT1^Cre^* mice. Briefly, *Snord3a^fl/fl^* and *Snord3a^fl/fl^‐GGT1^Cre^* mice were intraperitoneally injected with cisplatin (20 mg kg^−1^) or saline, and sacrificed at 3 days after administration. B) Kidney function assessed by serum creatinine and BUN in *Snord3a^fl/fl^* and *Snord3a^fl/fl^‐GGT1^Cre^* mice treated with cisplatin or saline (n = 9 for each group). C,D) Representative images of HE and PAS staining of renal sections from *Snord3a^fl/fl^* and *Snord3a^fl/fl^‐GGT1^Cre^* mice treated with cisplatin or saline, and quantification of tubular injury score (n = 9 for each group). Scale bars, 100 µm. E,F) Immunoblotting analysis and quantification of KIM1 and NGAL in *Snord3a^fl/fl^* and *Snord3a^fl/fl^‐GGT1^Cre^* mice treated with cisplatin or saline (n = 9 for each group). G,H) Representative immunofluorescence images of KIM1 (green), LTL (red), and NGAL (green) in *Snord3a^fl/fl^* and *Snord3a^fl/fl^‐GGT1^Cre^* mice treated with cisplatin or saline, and quantification of positive areas (n = 9 for each group). Scale bars, 100 µm. Data were presented as means ± SD. Statistical analysis was performed using one‐way ANOVA (B, D, F, and H). ^**^ *P* < 0.01, ^****^ *P* < 0.0001.

Having observed elevated levels of Snord3a in kidney tissues during AKI, we sought to investigate its role in AKI progression. To elucidate this, we generated *Snord3a*‐ knockout mice targeting renal tubular epithelium (*Snord3a^fl/fl^‐GGT1^Cre^* mice) (Figure S3A, Supporting Information), and all mice were genotyped by PCR analysis (Figure S3B, Supporting Information). Next, we established a cisplatin‐induced AKI model in both *Snord3a^fl/fl^‐GGT1^Cre^* mice and control *Snord3a^fl/fl^* mice (**Figure** **2**A), and observed a significant reduction in Snord3a levels through FISH staining and RT‐PCR analysis (Figure S3C, Supporting Information). The cisplatin‐treated *Snord3a^fl/fl^‐GGT1^Cre^* mice exhibited lower creatinine and blood urea nitrogen (BUN) compared to the cisplatin‐treated *Snord3a^fl/fl^* mice (Figure 2B). Notably, HE and PAS staining demonstrated that Snord3a knockout attenuated cisplatin‐induced tubular epithelial cell swelling, necrosis, shedding, and inflammatory cell infiltration (Figure 2C,D). To assess renal epithelial injury in the cisplatin‐induced AKI model, we conducted kidney injury molecule 1 (KIM1) and neutrophil gelatinase‐associated lipocalin (NGAL) to label injured tubules, along with LTL to mark healthy proximal tubules. Snord3a knockout was observed to recover the cisplatin‐induced increases in KIM1 and NGAL (Figure 2E–H).

To further determine the protective role of Snord3a deficiency in AKI progression, we established an IRI‐induced AKI model in *Snord3a^fl/fl^‐GGT1^Cre^* mice and *Snord3a^fl/fl^* mice (Figure S4A, Supporting Information), noting significant Snord3a knockdown efficiency through FISH staining and RT‐PCR analysis (Figure S3D, Supporting Information). Consistently, serum creatinine and BUN levels were dramatically lower in IRI‐induced *Snord3a^fl/fl^‐GGT1^Cre^* mice compared with IRI‐induced *Snord3a^fl/fl^* mice (Figure S4B, Supporting Information). Furthermore, Snord3a knockout protected the kidney against IRI‐induced progressive structural defects and tubular injury (Figure S4C–H, Supporting Information). These results demonstrate that Snord3a deficiency plays a protective role in kidney function and tubular injury in AKI mouse models.

### 2.3 Snord3a Accelerates Ferroptosis of AKI In Vitro and In Vivo

**Figure 3:**
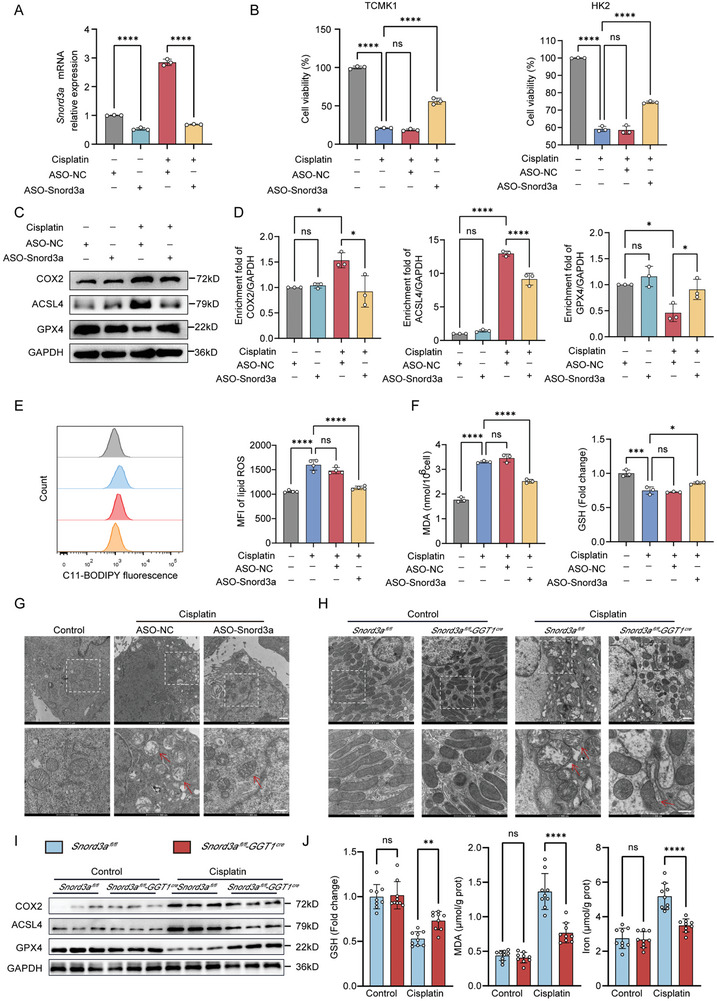
Snord3a accelerates ferroptosis of AKI in vitro and in vivo. A) The knockdown efficiency of Snord3a was evaluated in TCMK1 cells with Snord3a ASO transfection treated with cisplatin (20 µm, 24 h, n = 3 for each group). B) Cell viability was measured in TCMK1 and HK2 cells with Snord3a ASO transfection treated with cisplatin (20 µm, 24 h, n = 3 for each group). C–G) TCMK1 cells were transfected with control or Snord3a ASO for 24 h and then treated with cisplatin (20 µm) for 24 h. C,D) Immunoblotting analysis and quantification of indicators related to ferroptosis (COX2, ACSL4, and GPX4). n = 3 for each group. E) Lipid peroxidation was detected by flow cytometry using the BODIPY 581/591C11 probe (n = 4 for each group). F) MDA levels and GSH levels in different groups (n = 3 for each group). G,H) Representative mitochondrial morphology was visualized by transmission electron microscope (6500X, scale bar = 1 µm or 2 µm; 17500X, scale bar = 500 nm). Arrow: diminished or vanished mitochondria crista, or ruptured mitochondrial outer membrane. I) Immunoblotting analysis of ACSL4, COX2, and GPX4 in *Snord3a^fl/fl^* and *Snord3a^fl/fl^‐GGT1^Cre^* mice treated with cisplatin or saline. (J) GSH levels, MDA levels, and Iron levels of the kidney in *Snord3a^fl/fl^* and *Snord3a^fl/fl^‐GGT1^Cre^* mice treated with cisplatin (20 mg kg^−1^) or saline (n = 9 for each group). Data were presented as means ± SD. Statistical analysis was performed using one‐way ANOVA (A, B, D, E, F, and J). ^*^ *P* < 0.05, ^**^ *P* < 0.01, ^***^ *P* < 0.001, ^****^ *P* < 0.0001.

Given that ferroptosis is an early event triggering an inflammatory response in AKI,^[^ ^14^ ^]^ we investigated whether Snord3a plays a protective role in AKI by regulating tubular cell death through ferroptosis. Our initial exploration involved knockdown experiments using antisense oligonucleotide (ASO) targeting Snord3a in TCMK1 cells (**Figure** **3**A). Down‐regulation of Snord3a significantly improved the suppressed cell viability of both cisplatin‐treated TCMK1 and HK2 cells (Figure 3B). The protein abundance of the ferroptosis signaling pathway (COX2 and ACSL4) was increased in cisplatin‐treated TCMK1 cells and repressed by Snord3a knockdown (Figure 3C,D). Snord3a knockdown ameliorated cisplatin‐induced down‐regulation of glutathione peroxidase 4 (GPX4), a key regulator of lipid peroxidation in ferroptosis (Figure 3C,D), but did not lead to the restoration of the cisplatin‐induced apoptosis pathway (BAX and BCL2), necroptosis pathway (RIPK1 and RIPK3), and pyroptosis pathway (GSDMD), as shown in Figure S5A–E (Supporting Information). Thus, Snord3a may function as a regulator of ferroptosis in AKI.

To further determine the effects of Snord3a on ferroptosis during AKI, we investigated several ferroptosis phenotypes in TCMK1 cells. C11‐BODIPY 581/591 staining and malondialdehyde (MDA) assays revealed that cisplatin treatment markedly increased the lipid ROS levels, while these effects were reversed by Snord3a knockdown (Figure 3E,F). Glutathione (GSH) is a robust scavenger of lipid peroxidation products, which is the main limiting factor in the ferroptosis process.^[^ ^15^ ^]^ We further found that Snord3a knockdown protected against cisplatin‐induced GSH depletion (Figure 3F). As ferroptosis is characterized by the rupture of outer mitochondrial membrane, the disappearance of mitochondria crista, and vacuolization of mitochondria,^[^ ^16^ ^]^ we observed the structure of mitochondria via transmission electron microscopy (TEM). Our result showed that Snord3a knockdown attenuated the changes in mitochondria in cisplatin‐induced TCMK1 cells (Figure 3G). These experiments were repeated in HK2 cells, and the same results were confirmed in cisplatin‐treated HK2 cells (Figure S6A–G, Supporting Information). Additionally, we overexpressed Snord3a expression in cisplatin‐treated TCMK1 cells (Figure S7A, Supporting Information). Overexpression of Snord3a exacerbated the cisplatin‐induced reductions in cell viability and GSH levels, while increasing levels of reactive oxygen species (ROS) and lipid ROS (Figure S7B–F, Supporting Information).

Additionally, we evaluated ferroptosis phenotypes in TECs‐specific knockout mice of Snord3a via cisplatin and IRI‐induced AKI models. Snord3a knockout downregulated the expression of ACSL4 and COX2, and promoted the expression of GPX4 in cisplatin‐induced kidney tissues (Figure 3I). Moreover, cisplatin‐induced ferroptosis was ameliorated by Snord3a deficiency, manifesting as iron accumulation, MDA production, and GSH depletion (Figure 3J). Indeed, Snord3a knockout attenuated the characteristic changes of mitochondria in ferroptosis (Figure 3H). Consistently, these pro‐ferroptosis changes were all ameliorated by Snord3a knockout in the IRI mice (Figure S8A–H, Supporting Information). These data collectively indicate that Snord3a is a regulator of ferroptosis against AKI both in vitro and in vivo.

### 2.4 Snord3a Facilitates the Transcription Activity of STING

Having observed that Snord3a deletion or depletion mitigates ferroptosis in AKI, we next investigated the underlying mechanism by which Snord3a regulates AKI. Given that Snord3a is involved in the guidance of Fibrillarin (FBL)‐mediated RNA 2′‐O methylation (Nm),^[^ ^17^ ^]^ we explored whether Snord3a regulates ferroptosis via FBL‐dependent RNA Nm. RNA pull‐down assays showed the interaction between Snord3a and FBL (Figure S9A, Supporting Information). However, FBL knockdown did not reverse the suppressive effects on cell viability and the promotion effects of MDA accumulation and GSH depletion induced by Snord3a overexpression (Figure S9B–D, Supporting Information).

**Figure 4:**
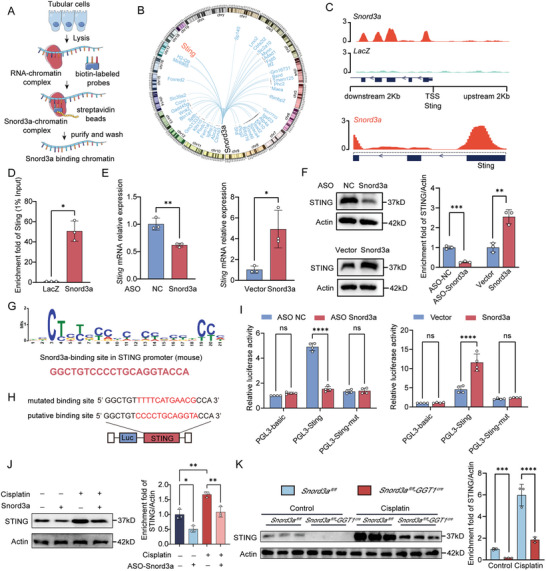
Snord3a facilitates the transcription of STING. A) Schematic diagram of identifying Snord3a binding chromatin by CHIRP. B) Circular plot showing interactions of top 50 genes enriched in the promoter region and Snord3a generated by CHIRP‐seq. C) Enrichment of chromatin binding sites for Snord3a at the promoter and gene body regions of STING detected by CHIRP‐seq. Bottom panels are a magnification of chromatin binding sites. D) CHIRP assay and RT‐qPCR analysis of the enrichment of STING (n = 3 for each group). E‐F) RT‐qPCR and immunoblotting analysis of STING in TCMK1 cells treated with Snord3a knockdown or overexpression (n = 3 for each group). G) Motif analysis of the binding peaks of Snord3a to STING promoter sequence based on JASPER website. H,I) Luciferase reporter assays were performed to assess the binding between STING and Snord3a in TCMK1 cells. The red letters indicate the putative or mutated Snord3a‐binding sequences. STING or binding site‐mutated STING was cloned into luciferase plasmids, and then the transduced TCMK1 cells co‐transfected with luciferase plasmid and Snord3a ASO, or luciferase plasmid and Snord3a plasmid (n = 4 for each group). J) Immunoblotting analysis and quantification of STING in TCMK1 cells with Snord3a ASO transfection treated with cisplatin (20 µm, 24 h, n = 3 for each group). K) Immunoblotting analysis and quantification of STING in *Snord3a^fl/fl^* and *Snord3a^fl/fl^‐GGT1^Cre^* mice treated with cisplatin or saline (n = 3 for each group). Data were presented as means ± SD. Statistical analysis was performed using one‐way ANOVA (F, I, J, and K) or Student's *t*‐test (D and E). ^*^ *P* < 0.05, ^**^ *P* < 0.01, ^***^ *P* < 0.001, ^****^ *P* < 0.0001.

Considering Snord3a's nuclear localization, it may exert a chromatin‐related function. To explore potential interactions between Snord3a and chromatin, we conducted chromatin isolation by RNA purification followed by DNA sequencing (CHIRP‐Seq) (**Figure** **4**A). After the pull‐down, Snord3a significantly increased in both biotinylated odd probe and even probe sets (Figure S10A, Supporting Information), and DNA was sequenced to identify potential Snord3a binding sites (Figure S10B, Supporting Information). Motif analysis suggested that Snord3a interaction with genomic DNA might happen through a distinct DNA motif (Figure S10C, Supporting Information). Furthermore, Snord3a motifs displayed significant similarity to several transcription factor‐binding sites previously identified (Figure S10C, Supporting Information), supporting the idea that Snord3a acts as a critical gatekeeper of the transcriptome. To identify candidate genes directly regulated by Snord3a, we analyzed the protein‐coding genes displaying at least one Snord3a‐binding site within their promoters. Among the potential binding promoters of candidate genes, STING ranked first with the highest tags (Figure 4B).

STING is known for its capacity to sense DNA and regulate innate immune activation, while growing evidence has linked it to the hallmark of ferroptosis.^[^ ^18^ ^]^ Indeed, the integrative genomics viewer showed the Snord3a binding site in the promoter region of *STING* gene (Figure 4C). Moreover, the CHIRP assay showed that Snord3a could significantly bind to STING chromatin (Figure 4D). Importantly, Snord3a positively regulated STING RNA and protein levels, which decreased significantly with Snord3a knockdown and increased after Snord3a overexpression in TCMK1 cells (Figure 4E,F). Next, we analyzed how Snord3a transcriptionally regulates STING. There was one putative motif for Snord3a binding to the STING promoter (Figure 4G). Indeed, dual‐luciferase assay confirmed that STING with a luciferase reporter plasmid containing the wild‐type sequence of Snord3a binding site significantly regulated the luciferase activities, while the mutant sequence did not alter the luciferase activities (Figure 4H,I). These results suggest that Snord3a is essential for the regulation of the *STING* gene transcription. We further confirmed that Snord3a not only regulated STING expression in cisplatin‐stimulated TCMK1 cells injury (Figure 4J), but also in cisplatin‐induced AKI mice model (Figure 4K).

### 2.5 Snord3a Interacts with STING to Promote Ferroptosis in AKI

**Figure 5:**
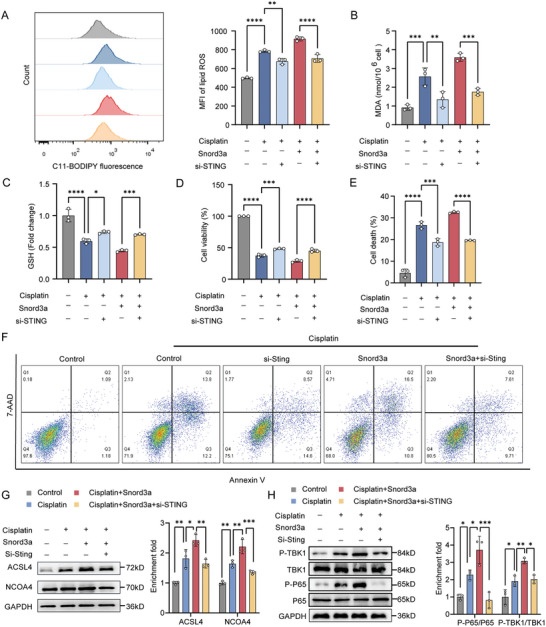
Snord3a interacts with STING to promote ferroptosis in AKI. A–H) TCMK1 cells were transfected with Snord3a plasmid or (and) STING siRNA before 24 h of cisplatin treatment (20 µm, 24 h). A) The lipid peroxidation level by BODIPY 581/591 C11 sensor (n = 3 for each group). B) MDA levels and C) GSH levels in different groups (n = 3 for each group). D) Cell viability was measured by CCK8 assay (n = 3 for each group). E‐F) Cell death was detected by flow cytometry using annexin V and 7‐AAD sensors. All relevant populations indicative of cell death, including Annexin V+ 7ADD‐, Annexin V+ 7ADD+, and Annexin V‐ 7ADD+ (n = 3 for each group). G,H) Immunoblotting analysis and quantification of ACSL4, NCOA4, and cGAS‐STING pathway (n = 3 for each group). Data were presented as means ± SD. Statistical analysis was performed using one‐way ANOVA (A, B, C, D, E, G, and H). ^*^ *P* < 0.05, ^**^ *P* < 0.01, ^***^ *P* < 0.001, ^****^ *P* < 0.0001.

Having established that Snord3a regulates the expression level of STING and is involved in the ferroptosis of AKI, we hypothesized that Snord3a may regulate the ferroptosis of AKI by controlling STING. Rescue experiments revealed that STING knockdown reversed the promotion effects of lipid ROS accumulation and GSH depletion induced by Snord3a overexpression (**Figure** **5**A–C). Meanwhile, STING knockdown abrogated the suppressive effects of cell viability and the stimulative effects of cell death elicited by Snord3a overexpression (Figure 5D–F). Conversely, the improvements of Snord3a knockdown in ferroptosis were counteracted by STING overexpression, as manifested by the increased ROS accumulation, lipid ROS accumulation, MDA production, GSH depletion, and reduced cell viability in cisplatin‐induced TCMK1 cells (Figure S12A–G, Supporting Information). These data suggest that Snord3a mediated the function of STING in ferroptosis.

Previous studies have demonstrated that STING induces ferroptosis in a manner dependent on its interaction with several effector molecules of ferroptosis, including nuclear receptor coactivator 4 (NCOA4) and ACSL4.^[^ ^19^ ^]^ We next investigated whether Snord3a regulates the key molecules of STING‐mediated ferroptosis in cisplatin‐stimulated TCMK1 cells. STING knockdown abrogated the promoting effects on ACLS4 and NCOA4 expression elicited by Snord3a overexpression (Figure 5G). Besides, we also observed the effects of Snord3a on the cGAS‐STING pathway. Notably, Snord3a overexpression accelerated the phosphorylation of the STING downstream target proteins (P65 and TBK1) by cisplatin stimulation, and these effects were markedly reversed by STING knockdown (Figure 5H). Taken together, these results indicate that Snord3a may activate the cGAS‐STING pathway, influence molecules of STING‐mediated ferroptosis, accelerate lipid peroxidation and GSH depletion, and finally promote ferroptosis.

**Figure 6:**
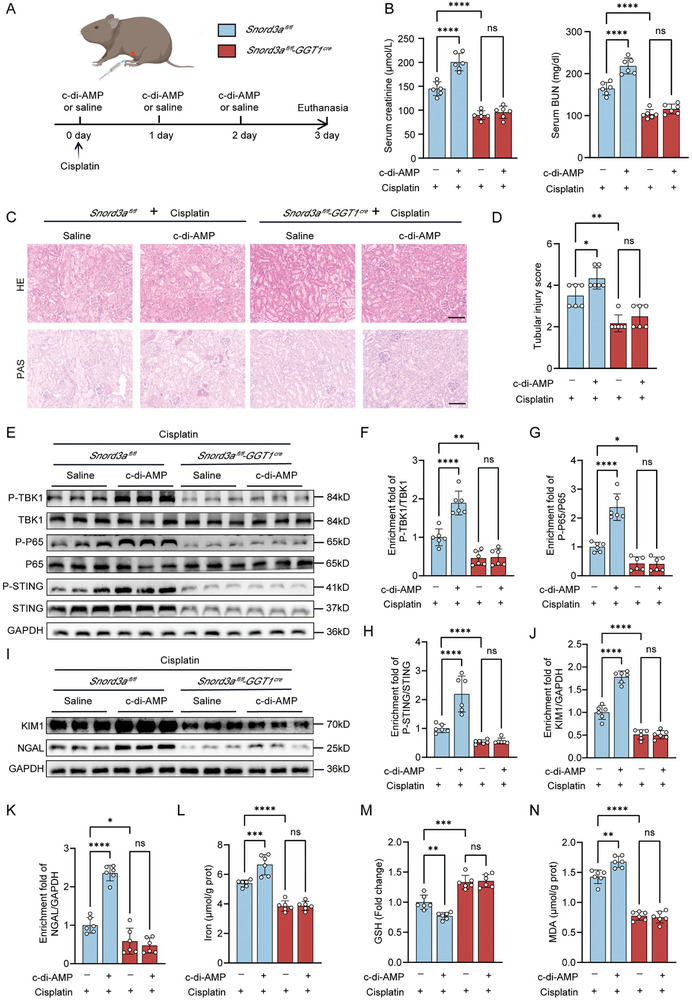
Snord3a deficiency alleviates renal injury aggravated by STING activation in cisplatin‐induced AKI model. A) Schematic representation illustrating the establishment of cisplatin‐induced AKI model with c‐di‐AMP or saline. Briefly, mice were intraperitoneally injected with c‐di‐AMP (25ug) or saline 1 h before cisplatin injection and continued once daily, and sacrificed at 3 days after administration. B) Kidney function was assessed by serum creatinine and BUN (n = 6 for each group). C,D) Representative images of HE and PAS staining of renal sections, and quantification of tubular injury score (n = 6 for each group). Scale bars, 100 µm. E–H) Immunoblotting analysis and quantification of cGAS‐STING pathway (n = 6 for each group). I–K) Immunoblotting analysis and quantification of KIM1 and NGAL (n = 6 for each group). L–N) Iron levels, GSH levels, and MDA levels of the kidney (n = 6 for each group). Data were presented as means ± SD. Statistical analysis was performed using one‐way ANOVA (B, D, F, G, H, J, K, L, M and N). ^*^ *P* < 0.05, ^**^ *P* < 0.01, ^***^ *P* < 0.001, ^****^ *P* < 0.0001.

Furthermore, we investigated the impact of STING activation on ferroptosis phenotypes in cisplatin‐induced AKI model among *Snord3a^fl/fl^‐GGT1^Cre^* mice and *Snord3a^fl/fl^* mice (**Figure** **6**A). The serum creatinine and BUN levels were significantly higher in c‐di‐AMP ‐treated *Snord3a^fl/fl^* mice compared with saline‐treated *Snord3a^fl/fl^* mice; however, c‐di‐AMP treatment unable to exacerbate renal dysfunction in *Snord3a^fl/fl^‐GGT1^Cre^* mice (Figure 6B). Notably, c‐di‐AMP treatment aggravated cisplatin‐induced renal epithelial cell swelling and detachment of cell necrotic tubules in *Snord3a^fl/fl^* mice, while Snord3a knockout attenuated c‐di‐AMP ‐induced structural defects and tubular injury (Figure 6C,D). The abundance of phosphorylated proteins downstream of the STING signaling pathway (TBK1 and P65) was increased during c‐di‐AMP treatment and downregulated by Snord3a knockout (Figure 6E–H). Moreover, Snord3a knockout recovered the up‐regulation of tubular injury indicators (KIM1 and NGAL) after c‐di‐AMP treatment (Figure 6I–K). As anticipated, the enhancement of ferroptosis by c‐di‐AMP was mitigated by the knockout of Snord3a, evidenced by reductions in iron accumulation, MDA production, and GSH depletion (Figure 6L–N). These results indicate that Snord3a knockout ameliorates c‐di‐AMP treatment‐induced renal dysfunction, tubular injury, and ferroptosis in cisplatin‐induced AKI.

### 2.6 Snord3a Serves as a Promising Therapeutic Target for AKI

**Figure 7:**
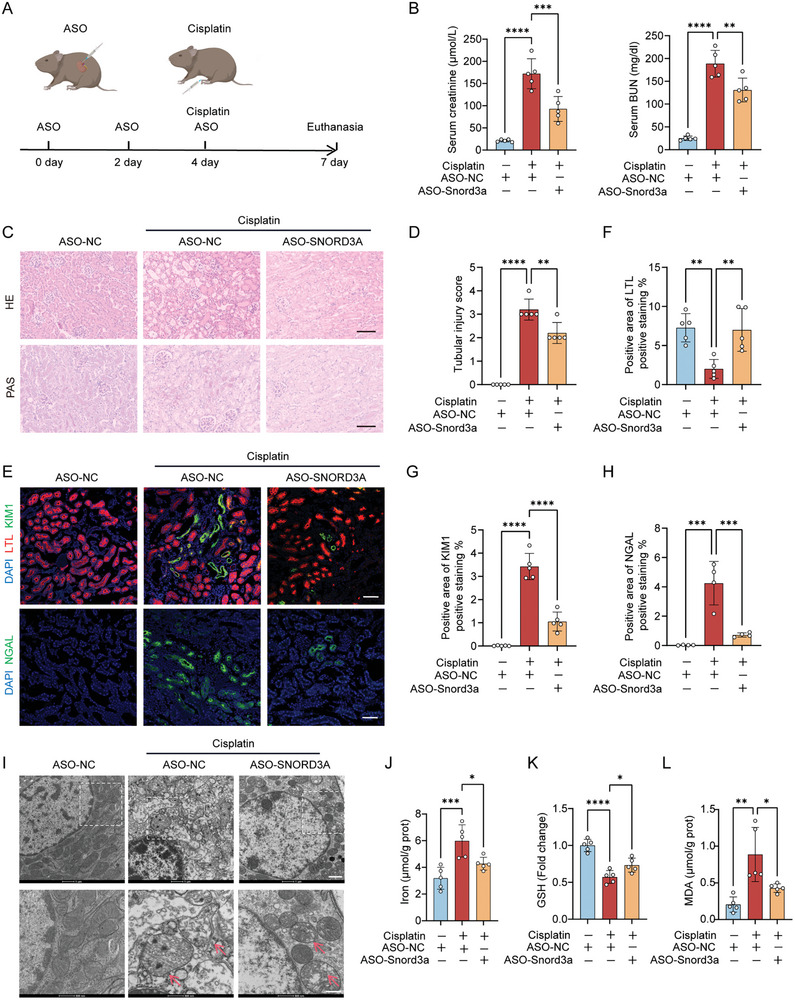
The therapeutical application of Snord3a ASO in cisplatin‐induced AKI mice model. A) Schematic diagram of Snord3a ASO treatment in the cisplatin‐induced AKI mice model. Briefly, each group was injected with the Snord3a ASO or negative control ASO in the mouse kidneys every two days for three times, and a cisplatin‐induced AKI mice model was performed after the ASO injection. B) Kidney function was assessed by serum creatinine and BUN levels (n = 5 for each group). C,D) Representative images of HE and PAS staining of renal sections, and quantification of tubular injury score (n = 5 for each group). Scale bars, 100 µm. E–H) Representative immunofluorescence images of KIM1 (green, n = 5 for each group) and co‐stained with LTL (red), or NGAL (green, n = 4 for each group), and quantification of positive areas. Scale bars, 100 µm. I) Representative mitochondrial morphology was visualized by transmission electron microscope (6500X, scale bar = 1 µm; 17500X, scale bar = 500 nm). Arrow: diminished or vanished mitochondria crista, or ruptured mitochondrial outer membrane. J‐L) Iron levels, GSH levels, and MDA levels of the kidney (n = 5 for each group). Data were presented as means ± SD. Statistical analysis was performed using one‐way ANOVA (B, D, F, G, H, J, K, and L). ^*^ *P* < 0.05, ^**^ *P* < 0.01, ^***^ *P* < 0.001, ^****^ *P* < 0.0001.

The important regulatory functions of Snord3a in AKI open avenues for exploring Snord3a as a potential therapeutic target. RNA‐based therapeutics hold tremendous promise for combating diseases that were previously challenging to treat.^[^ ^20^ ^]^ Therefore, an antisense oligonucleotide targeting Snord3a was rationally designed and synthesized to down‐regulate the expression of Snord3a through the renal injection. To validate the clinical application of Snord3a in vivo, the cisplatin‐induced and IRI‐induced AKI mice were injected with the Snord3a ASO or control ASO in the mouse kidneys every two days for three times (**Figures** **7**A and S14A, Supporting Information). The results revealed that the serum creatinine and BUN levels were significantly restored in Snord3a ASO‐treated AKI compared to the control ASO group (Figure 7B and Figure S14B, Supporting Information). Significantly downregulated Snord3a expression in the kidney tissues was observed in Snord3a ASO‐treated AKI by FISH staining and RT‐PCR analysis (Figures S13A,B and S15A,B, Supporting Information). Moreover, Snord3a ASO treatment protected the kidney against cisplatin‐induced and IRI‐induced progressive structural defects and tubular injury (Figure 7C,D and Figure S14C,D, Supporting Information). Indeed, Snord3a ASO treatment recovered the up‐regulation of KIM1 and NGAL, and upregulated LTL levels in AKI (Figure 7E–H; Figures S13C–E, S14E–H and S15C–G, Supporting Information).

Furthermore, we explored the role of Snord3a ASO attenuated ferroptosis in AKI. As TEM analysis showed the characteristic changes of mitochondria in ferroptosis were ameliorated in Snord3a ASO‐treated AKI (Figure 7I and Figure S14I, Supporting Information). Notably, both cisplatin‐induced and IRI‐induced ferroptosis were improved by Snord3a ASO treatment, manifesting as iron accumulation, GSH depletion, and MDA production (Figure 7J–L; Figure S14J–L, Supporting Information). A significant therapeutic advantage is established with Snord3a ASO treatment in the present study, which indicates Snord3a may serve as a potential therapeutic target in AKI.

## 3 Discussion

This study unveils a Snord3a‐mediated mechanism in accelerating AKI progression through ferroptosis. We found the heightened expression of Snord3a in TECs during AKI, emphasizing its involvement in driving tubular ferroptosis by activating the STING pathway. Snord3a enhances STING expression through the promotion of STING gene transcription. Notably, ASOs targeting Snord3a emerge as a promising therapeutic strategy for AKI. These findings not only highlight the potential of Snord3a as a novel indicator and therapeutic target for AKI but also contribute to a broader understanding of snoRNA function mechanisms.

Studies have reported high expression of Snord3a in breast cancer, osteosarcoma, and hepatocellular carcinoma, where it serves as a diagnostic molecular marker.^[^ ^21^ ^]^ Liu et al. observed that Snord3a enhanced cell proliferation and reprogrammed energy metabolism in glioblastoma.^[^ ^22^ ^]^ Moreover, Tim et al. indicated that impaired expression of Snord3a in osteoarthritis affected chondrocyte phenotype and inflammation by regulating translation capacity.^[^ ^23^ ^]^ Despite these findings, the biological role and molecular functions of Snord3a in kidney diseases have remained elusive. Our study unveiled a significant upregulation of Snord3a in both murine and human tubular epithelial cells during AKI. Moreover, the human subject study indicated that renal Snord3a concentration negatively correlates with renal function, suggesting the pathological role of Snord3a in kidney disease.

Accumulating evidence demonstrates the predominance of ferroptosis in promoting tubular injury in the AKI process. Of particular significance, the continuous stimulation of iron and lipid peroxidation in ferroptosis further provokes cell death on an expanding scale, thereby exacerbating renal injury.^[^ ^12^ ^]^ ACSL4, an enzyme that converts fatty acids to fatty acyl‐CoA esters, regulates lipid peroxides. Gao et al. revealed that ACSL4 knockout prevented the accumulation of lipid peroxides, thus alleviating ferroptotic signal propagation and kidney injury in AKI.^[^ ^24^ ^]^ Furthermore, the GSH/GPX4 axis serves as a robust scavenger of lipid peroxidation products, representing a critical limiting factor in the ferroptosis process. Conrad et al. also elucidated the essential role of the GSH/GPX4 axis in preventing lipid‐oxidation‐induced acute renal failure and ferroptotic cell death using inducible GPX4(‐/‐) mice.^[^ ^25^ ^]^ Our research suggests that Snord3a inhibits the normal biology of GPX4 and ACSL4 in lipid metabolism, leading to lipid peroxides accumulation and GSH depletion. Remarkably, Snord3a deficiency not only disrupted the ferroptosis process but also mitigated kidney damage, underscoring the critical role of Snord3a‐mediated ferroptosis in defending against AKI. Snord3a deficiency partially ameliorated kidney damage during AKI, primarily due to Snord3a's regulation of the AKI process being limited to ferroptosis. However, AKI involves various types of TEC death beyond ferroptosis, including apoptosis, necroptosis, and pyroptosis. Multiple studies have demonstrated the prominence of apoptosis in the setting of AKI, and deletion of apoptosis‐associated proteins such as caspase‐8 or Fas from renal tubules has protected AKI.^[^ ^26^ ^]^ Necroptosis is mediated by RIPK‐dependent phosphorylation of the MLKL and subsequent plasma membrane rupture, which is an important mediator of tubular injury. MLKL is among the most significantly upregulated genes in various models of AKI, and RIPK3 and MLKL deficient mice have shown protection from the IRI model.^[^ ^27^ ^]^ Recently, studies have shown that GSDMD plays a protective role in renal tubules by preventing necroptosis‐mediated damage during the AKI process. In IRI and cisplatin‐induced AKI, GSDMD‐deficient mice exhibited increased sensitivity to injury, as evidenced by tubular damage and elevated serum creatinine levels.^[^ ^28^ ^]^

STING is an adaptor protein involved in DNA‐dependent activation of the innate immunity in autoinflammatory diseases.^[^ ^29^ ^]^ Growing evidence has connected STING to GPX4‐mediated lipid peroxidation within the context of ferroptosis.^[^ ^30^ ^]^ Our recent findings demonstrated that STING plays a regulatory role in inducing ferroptosis in TECs during ischemic AKI, and STING deficiency alleviated renal ischemia‐reperfusion injury through the process of ferritinophagy.^[^ ^19b^ ^]^ In the present study, we provide evidence that the knockout of Snord3a mitigates renal dysfunction, tubular injury, and ferroptosis induced by STING activation in cisplatin‐induced AKI. This implies a crucial involvement of Snord3a in the activation of the cGAS‐STING pathway during AKI. Notably, our data suggest that Snord3a can bind significantly to STING chromatin, thereby promoting the transcription of the STING gene. Further refinement is needed to elucidate the intricacies of the transcriptional regulation of STING by Snord3a.

Box C/D snoRNAs are essential players in RNA modifications due to their involvement in the guidance of RNA Nm. FBL serves as the key mediator of RNA Nm modifications, guided by Box C/D snoRNAs, thereby influencing gene expression by modulating mRNA levels and protein translation.^[^ ^17^ ^]^ Although our research uncovers the reciprocal binding between Snord3a and FBL, the exacerbated ferroptosis mediated by Snord3a overexpression is not rescued by FBL knockdown. However, further investigation is necessary to elucidate whether Nm‐induced RNA modifications also contribute to Snord3a‐associated ferroptosis in AKI.

Overall, our study elucidated the underlying mechanism of AKI in a Snord3a/STING‐dependent manner. In this study, we highlight RNA therapy targeting Snord3a ASOs as a promising therapeutic strategy for alleviating ferroptosis and kidney damage in AKI. Importantly, our findings revealed a detrimental role of hyperactive STING signaling in promoting ferroptosis and inflammation in AKI, suggesting the potential for interventions targeting STING signaling to enhance the efficacy of conventional treatments.

## 4 Experimental Section

### Cell Culture and Intervention

Tubular epithelial cell lines (HK2 and TCMK1) were cultured in RPMI 1640 medium supplemented with 10% fetal bovine serum and 1% penicillin and streptomycin in the incubator at 37°C with 5% CO2. All cell lines were authenticated through repeat profiling and confirmed to be mycoplasma‐free.

Snord3a and STING plasmids were synthesized by RealGene Bio‐tech and respectively cloned into the PLVX‐puro vector tagged with a flag. Snord3a ASO and negative control ASO were designed from RIBOBIO, while the siRNAs of Sting, FBL and the negative control were purchased from GenePharma. The sequence details were listed in Supplementary Table S4 (Supporting Information). Cell transfection with plasmid, ASO, or siRNA was performed by using Lipofectamine 3000 (L3000‐015, Invitrogen) according to the manufacturer's protocols. For specific experiments, TCMK1 and HK2 cells were treated with cisplatin (S1166, Selleck).

### Animal Experiments

*Snord3a^fl/fl^* mice were generated through a gene targeting method (GemPharmatech). Subsequently, *Snord3a^fl/fl^* mice were crossbred with *GGT1^Cre^* mice to generate heterozygous mice and obtain *Snord3a^fl/fl^‐GGT1^Cre^* mice (conditional knockout of Snord3a in mouse kidney epithelial cells) and *Snord3a^fl/fl^* mice (control).

For cisplatin‐induced AKI models, male mice aged 8 to 10 weeks received a single intraperitoneal injection of 20 mg kg^−1^ cisplatin. The mice were euthanized at 72 h, and serum samples and kidney tissues were collected for further analysis. For IRI‐induced AKI models, the unilateral renal vessel was clamped with a vascular clip for 30 min at 37 °C, as described previously.^[^ ^31^ ^]^ The same procedure without renal vessel clamps was performed in the sham group. The mice were sacrificed 24 h later, and serum samples and kidney tissues were collected for further analysis. Renal function was monitored by determining serum creatinine and BUN using an automatic dry‐chemistry analyzer (FUJIFLIM, DRI CHEM 7000i).^[^ ^32^ ^]^

For treatment with c‐di‐AMP (MCE, HY‐12326A) in vivo, mice were intraperitoneally injected with c‐di‐AMP (25ug) or saline 1 h before cisplatin injection, and injections were continued once daily. Mice were euthanized at 72 h after cisplatin injection, and serum samples and kidney tissues were collected for further analysis.

For treatment with Snord3a ASO in vivo, each group received internal injections of Snord3a ASO (5 nmol) or control ASO (5 nmol) every two days for 3 times. Briefly, the left kidney of the mouse was exposed, and 5 nmol ASO was multipoint injected using a 33G micro‐syringe (Hamilton) with an injection depth of ≈ 1–2 mm (corresponding to the corticomedullary junction of the kidney). AKI models were performed after ASO injection.

All animal experiments were approved by the Animal Ethics Committee of Zhejiang University (Approval No. 20231479).

### Human Samples

A total of 30 patients diagnosed with AKI and 30 appropriately matched healthy donors were enrolled from the Kidney Disease Center of the First Affiliated Hospital of Zhejiang University. The inclusion criteria for AKI patients were as follows: 1) confirmation of AKI based on the Kidney Disease Improving Global Outcome;^[^ ^33^ ^]^ 2) validation of AKI in accordance with renal pathology; and 3) patients aged between 18 and 80 years. Healthy donors exhibited no signs of disease, and the detailed clinical information was listed in Supplementary Table S3 (Supporting Information). The study received approval from the ethical committee of the First Affiliated Hospital of Zhejiang University based on the Declaration of Helsinki (Approval No. 20240024). All subjects provided the consent for their participation in this study.

### Immunoblotting

Cells or kidney tissues were lysed using RIPA buffer supplemented with protease and phosphatase inhibitors, and the protein concentration was measured by a Bradford assay (P0006C, Beyotime). Cell lysates were subjected to SDS–PAGE, and then transferred to PVDF membranes. After blocking, the membranes were incubated with primary antibodies overnight at 4 °C. On the next day, the membranes were washed and incubated with corresponding secondary antibodies for 1 h at room temperature. The antibodies were listed in Table S5 (Supporting Information).

### Real‐Time PCR

Total RNA was extracted from cells or kidney tissues using the Steady Pure Quick RNA Extraction Kit (AG21023, Accurate Biology), followed by cDNA synthesis with Evo M‐MLV RT Mix Kit (AG11728, Accurate Biology). RT‐PCR was performed with SYBR Green Premix Kit (AG11701, Accurate Biology) and ran on a CFX96 instrument (Bio‐Rad Laboratories). The primer details were listed in Table S6 (Supporting Information).

### Cell Viability Assay

Cell viability was assessed using a Cell Counting Kit‐8 (HY‐K0301, MedChemExpress). In brief, cells were seeded into 96‐well plates at a density of 4 ×10^3^ per well. The following day, cells were treated with cisplatin (20 µm) for 24 h. Subsequently, cells were exposed to 10 µl CCK‐8 reagent (100 µl medium per well), and incubated for 2 h at 37 °C in a 5% CO2 incubator. The absorbance of each well was measured at a wavelength of 450 nm.

### Cell Death Assay

Cell death was evaluated by flow cytometry after treatment with cisplatin (20 µm) for 24 h. Cells were stained with 7‐Amino‐Actinomycin D (7‐AAD) and Annexin V for 15 min at room temperature without light. Subsequently, 1 × 10^4^ cells per sample were investigated by flow cytometry (BD Biosciences), and the percentage of the death cells was analyzed by the FlowJo software (v10.4 BD). All relevant populations indicative of cell death, including Annexin V+ 7ADD‐, Annexin V+ 7ADD+, and Annexin V‐ 7ADD+.

### ROS Measurement

Cells were planted into 12‐well plates and incubated overnight. The next day, cells were treated with cisplatin (20 µm) for 24 h. Subsequently, cells were stained with 2 µm Rhodamine 123 (D1054, Sigma) for 15 min at 37 °C in a 5% CO2 incubator. Afterward, cells were washed and resuspended in PBS. More than 1 × 10^4^ cells per sample were assessed by flow cytometry with a 488 nm laser, and data were analyzed by the FlowJo software.

### Lipid Peroxidation Measurement

Cells were planted into 12‐well plates and incubated overnight. The following day, cells were treated with cisplatin (20 µm) for 24 h. Subsequently, cells were stained with 5 µm C11‐BODIPY 581/591 (D3861, Thermo Fisher Scientific) for 30 min at 37 °C in a 5% CO2 incubator. After staining, cells were washed thrice with PBS to remove the excess labeling mixture, and 1 × 10^4^ cells per sample were assessed by flow cytometry. Lipid peroxidation resulted in a shift of the fluorescence emission peak from 590 to 510 nm, and data were analyzed by the FlowJo software.

### MDA Measurement

The concentration of MDA was determined using the Lipid Peroxidation MDA Assay kit (ab118970, Abcam). Briefly, 200 µl of cells or kidney tissues sample was mixed with 600 µl of MDA working solution and incubated at 95°C for 1 h. After centrifugation, 200 µl of the reaction mix was transferred into a 96‐well microplate, and the absorbance of each sample was immediately measured at 532 nm on a microplate reader.

### GSH Measurement

Cells or kidney tissues were collected and prepared for measurement of GSH using the Glutathione Assay Kit (S0053, Beyotime Biotechnology) according to the provided instructions. In short, 100 µl of the sample was mixed with a GSH working solution, and incubated at 25 °C for 1 h to assess the GSSG level. Then, the above sample was incubated with the total glutathione working solution, and the absorbance was measured at 412 nm on a microplate reader. The GSH and GSSG concentrations were calculated using a standard curve and normalized to the total protein level.

### Iron Measurement

Intracellular ferrous iron (Fe^2+^) levels in kidney tissues were assessed using the Iron Assay Kit (ab83366, Abcam) according to the instructions. Briefly, 100 µl of the sample was mixed with 5 µl of iron buffer and incubated at 37 °C for 30 min in 96‐well plates. Subsequently, 100 µl of the iron probe was added into each well and incubated at 37 °C for 1 h protected from light. Finally, the absorbance was measured at 593 nm on a microplate reader.

### Transmission Electron Microscopy

Cells and kidney tissues were collected and immediately fixed in glutaraldehyde at 4°C. After post‐fixed with osmium tetroxide, samples were dehydrated through a graded ethanol series. Subsequently, the samples were embedded and polymerized at 37°C overnight. The obtained samples were cut into 80 nm ultrathin sections using an ultra‐thin microtome. After staining with uranium acetate and lead citrate, images were acquired by TEM (Talos 120 kV).

### Histology and Immunofluorescence

Fresh kidney tissues were fixed with 4% paraformaldehyde overnight, washed with PBS, and stored in 75% ethanol at 4 °C. After dehydration and embedding, the obtained samples were cut into 8 µm thickness for HE or PAS staining. Tubular damage was evaluated based on the following parameters: tubular brush border loss, tubular dilatation and disruption, flattened epithelial cells, and sloughing of tubular epithelial cells. The quantification of tubular damage was assessed in a blinded manner and scored based on the percentage of injured tubules: 0, no damage; 1, <25%; 2, 25%–50%; 3, 50%–75%; 4, >75%.

For immunofluorescence, the frozen sections were permeabilized with 0.1% Triton X‐100 and blocked with 5% BSA at room temperature for 1 h, and then incubated with primary antibodies at 4°C overnight. Subsequently, sections were incubated with secondary antibodies at room temperature for 1 h protected from light. After incubation, sections were counterstained with DAPI at room temperature for 10 min and mounted and coverslipped with an antifade mounting medium. Images were captured by the confocal microscope (Nikon A1 Ti).

### Fluorescence In Situ Hybridization

Snord3a probes were synthesized and utilized with BaseScope Reagent Kit (323900‐USM, Advanced Cell Diagnostics). Briefly, cells and kidney tissues were incubated with RNA Scope H_2_O_2_ and protease in turn. Then sections with probes inside the hybridization oven for 2 h at 40 °C, followed by hybridization with BaseScope AMP1–8 one by one. Finally, the signals were detected using BaseScope Fast RED, and nuclei were stained with hematoxylin dye or DAPI. Images were captured by the confocal microscope (Nikon A1 Ti). Positive signals were identified by red dot or cluster signals.

### RNA Pull‐Down Assay

The biotin‐labeled probes targeting Snord3a and negative probes were directly synthesized by Tsingke Biotech. The biotin‐labeled probes were incubated with the Streptavidin beads (11205D, Thermo Fisher Scientific) for 30 min at room temperature rotating all the time. Next, cell lysates were employed for incubation with probe‐coated beads for 2 h at 4 °C to pull down the RNA‐binding proteins. Finally, the RNA‐binding proteins were eluted sequentially and analyzed by immunoblotting. The detailed information of the sequences was shown in Table S4 (Supporting Information).

### Chromatin Isolation by RNA Purification (CHIRP)

The DNA biotin‐labeled probes targeting Snord3a and negative probes were directly synthesized by Sangon Biotech. In brief, 2 × 10^7^ cells were fixed with 3% formaldehyde solution for 15 min at room temperature, quenched by glycine, and followed by ultrasonic lysis to 200–500 bp. The biotin‐labeled probes were incubated with the chromatin complex in a hybridization buffer for 4 h at 37°C rotating all the time. Then, streptavidin beads (11205D, Thermo Fisher Scientific) were added to immunoprecipitate the chromatin complex for 1 h at 37°Crotating all the time. DNA was eluted sequentially and de‐crosslinked to prepare for CHIRP‐seq. The sequences of probes were listed in Table S4 (Supporting Information).

### Dual‐Luciferase Reporter Assay

Sting promoter region, as well as the candidate mutated sequence, were inserted into a PGL3‐basic vector (Promega). All constructed target fragments were confirmed by sequencing. TCMK1 cells were co‐transfected by luciferase reporter vector and renilla vector (Internal control) under Snord3a knockdown or overexpression. Luciferase activities were examined using the Dual‐Luciferase Reporter Assay System (Promega, E1910) and measured by a microplate reader.

### Statistical Analysis

The data were presented as means ± standard deviations (SD) from at least three biological replicates of experiments. The differences between groups were assessed using Student's *t*‐test and one‐way ANOVA. *P* < 0.05 was considered statistically significant. All statistical analysis was performed by the GraphPad Prism software and the Statistical Program for Social Sciences software.

## Conflict of Interest

The authors declare no conflict of interest.

## Author Contributions

H.Z., and J.W., contributed equally to this work. F.H., and W.L., conceived and designed the research. H.Z., and J.W., performed most of the biochemical and molecular experiments and bioinformatics analysis, with assistance from J.M., M.S., X.H., H.W., A.N., and H.Z., and J.W., performed in vivo experiments. H.Z., performed bioinformatics analysis. H.Z., contributed to the clinical sample collection. F.H., W.L., J.C., L.X., and S.X., provide experimental guide. H.Z., and J.W., analyzed the data and wrote the manuscript. F.H., W.L., and S.X., supervised this research and edited the paper.

## Supporting information

Supporting Information

## Data Availability

The data that support the findings of this study are available from the corresponding author upon reasonable request.
