# Supplementary material for: SNORD3A Regulates STING Transcription to Promote Ferroptosis in Acute Kidney Injury
Source: Adv Sci (Weinh). 2024 Jul 4;11(33):2400305. doi: 10.1002/advs.202400305 (PMC11434033; doi:10.1002/advs.202400305)
Supplement: Supplementary file 1 — Supporting Information [file ADVS-11-2400305-s001.docx]

**SNORD3A Regulates STING Transcription to Promote Ferroptosis in Acute Kidney Injury**

Huanhuan Zhu^1†^, Junni Wang^1†^, Jin Miao^1^, Mingdi Shen^1^, Huijing Wang^1^, Xiaohan Huang^1^, Anqi Ni^1^, Huijuan Wu^3^, Jianghua Chen^1^, Liang Xiao^1^, Shanshan Xie^4^, Weiqiang Lin^2*^, Fei Han^1*^

1 Kidney Disease Center, The First Affiliated Hospital, Zhejiang University School of Medicine; Institute of Nephrology, Zhejiang University; Key Laboratory of Kidney Disease Prevention and Control Technology, Zhejiang Province; Zhejiang Clinical Research Center of Kidney and Urinary System Disease, Hangzhou 310003, China

2 The Fourth Affiliated Hospital of School of Medicine, and International School of Medicine, International Institutes of Medicine, Zhejiang University, Yiwu 322000, China

3 Department of Pathology, School of Basic Medical Sciences, Fudan University, Shanghai 200032, China

4 Children’s Hospital, Zhejiang University School of Medicine, National Clinical Research Center for Child Health, Hangzhou 310052, Zhejiang, China

†These authors contributed equally to this work.

*Corresponding authors.

Email: hanf8876@zju.edu.cn; wlin@zju.edu.cn.

**This file includes:**

Figure. S1 to S16

Table. S1 to S6

**Supplementary Figures**

**Fig. S1. Increased snoRNAs expression in AKI.** (A) Volcano plot of snoRNAs expression in TCMK1 cell treated with cisplatin (20 μM, 24h) versus untreated TCMK1 cells (n=3 for each group). (B) Heat map of the snoRNAs that were up-regulated expression more than twofold (n=3 for each group). (C-D) RT-qPCR analysis of the 9 up-regulated snoRNAs expression in TCMK1 and HK2 cells treated with cisplatin (20 μM, 24h). n=4 for the TCMK1 cells group and n=3 for the HK2 cells group. Data were presented as means ± SD. Two-sided Student’s t test is used for (C, D). **P* < 0.05, ***P* < 0.01, *****P* < 0.0001.

**Fig. S2. Cisplatin-induced and IRI-induced kidney injury mice models.** (A) Schematic diagram of cisplatin-induced AKI mouse model. Briefly, mice were intraperitoneal injected with cisplatin (20mg/kg) or saline, and sacrificed at 3 days after administration. (B) Kidney function of serum creatinine and BUN in cisplatin-induced AKI at indicated time points (n=3 for each group). (C) Schematic diagram of IRI-induced AKI mouse model. (D) Kidney function of serum creatinine and BUN in IRI-induced AKI at indicated time points (n=4 for each group). (E-F) RT-qPCR analysis *of Kim1 and Lcn2* mRNA in mice with cisplatin-induced kidney injury (n=3 for each group) and IRI-induced kidney injury (n=4 for each group) at indicated time points. Data were presented as means ± SD. One-way ANOVA test is used for (B, D, E, and F). **P* < 0.05, ***P* < 0.01, ****P* < 0.001, *****P* < 0.0001.

**Fig. S3. Validation and expression of Snord3a in AKI models of Snord3a knockout mice.** (A) Schematic diagram illustrating the genetic approach used to generate Snord3a knockout mice targeted to the renal tubular epithelium. (B) Genomic DNA assessment of the mice genotype. (C) FISH and RT-qPCR analysis of Snord3a expression in *Snord3a^fl/fl^* and *Snord3a^fl/fl^-GGT1^Cre^* mice treated with cisplatin or saline. Scale bars, 100 μm. n=3 for each FISH group and n=9 for each RT-qPCR group. (D) FISH and RT-qPCR analysis of Snord3a expression in *Snord3a^fl/fl^* and *Snord3a^fl/fl^-GGT1^Cre^* mice treated with sham or IRI surgery. Scale bars, 100 μm. n=3 for each FISH groups and n=6 for each RT-qPCR groups. Data were presented as means ± SD. One-way ANOVA test is used for (C, D). *****P* < 0.0001.

**Fig. S4. Snord3a deficiency alleviates renal injury in IRI-induced AKI model.** (A) Schematic representation illustrating the establishment of IRI-induced AKI model in *Snord3a^fl/fl^* and *Snord3a^fl/fl^-GGT1^Cre^* mice. (B) Kidney function of serum creatinine and BUN in *Snord3a^fl/fl^* and *Snord3a^fl/fl^-GGT1^Cre^* mice treated with sham or IRI surgery (per group n=6). (C-D) Representative images of HE and PAS staining of renal sections from *Snord3a^fl/fl^* and *Snord3a^fl/fl^-GGT1^Cre^* mice treated with sham or IRI surgery, and quantification of tubular injury score (per group n=6). Scale bars, 100 μm. (E-F) Immunoblotting analysis and quantification of KIM1 and NGAL in *Snord3a^fl/fl^* and *Snord3a^fl/fl^-GGT1^Cre^* mice treated with sham or IRI surgery (per group n=6). (G-H) Representative immunofluorescence images of KIM1 (green), LTL (red), and NGAL (green) in *Snord3a^fl/fl^* and *Snord3a^fl/fl^-GGT1^Cre^* mice treated with sham or IRI surgery, and quantification of positive areas (per group n=6). Scale bars, 100 μm. Data were presented as means ± SD. One-way ANOVA test is used for (B, D, F, and H). **P* < 0.05, ***P* < 0.01, *****P* < 0.0001.

**Fig. S5. Functions of Snord3a in regulated cell death pathways.** TCMK1 cells were transfected with control ASO or Snord3a ASO before 24 h of cisplatin treatment (20 μM, 24h). (A-C) Immunoblotting analysis and quantification of RIPK1, RIPK3, BAX, and BCL2 protein expression (n=3 for each group). (D-E) Immunoblotting analysis and quantification of GSDMD expression (n=3 for each group). Data were presented as means ± SD. One-way ANOVA test is used for (B, C, and E). **P* < 0.05, ****P* < 0.001.

**Fig. S6. Snord3a deficiency ameliorates ferroptosis in cisplatin-treated HK2 cells.** HK2 cells were transfected with control ASO or Snord3a ASO before 24h of cisplatin treatment (20 μM, 24h). (A) The knockdown efficiency of Snord3a was evaluated by qRT-PCR analysis in HK2 cells (n=3 for each group). (B) Cell viability was measured by CCK8 assay (n=3-4 for group). (C) GSH levels and (D) MDA levels in different groups (n=3 for each group). (E-F) Intracellular ROS and lipid peroxidation were detected by flow cytometry using a DHR123 probe and a BODIPY 581/591C11 probe (n=3 for each group). (G) Representative mitochondrial morphology was visualized by transmission electron microscope (6500X, scale bar = 1 μm; 17500×, scale bar = 500 nm). Arrow: diminished or vanished mitochondria crista, or ruptured mitochondrial outer membrane. Data were presented as means ± SD. One-way ANOVA test is used for (A, B, C, D, E, and F). **P* < 0.05, ***P* < 0.01, ****P* < 0.001, *****P* < 0.0001.

**Fig. S7. Snord3a overexpression exacerbates ferroptosis in cisplatin-treated TCMK1 cells.** TCMK1 cells were transfected with empty vector plasmid (Vector) or Snord3a overexpression plasmid (Snord3a), and then the cells were either untreated or treated with cisplatin (20 μM) for 24h. (A) The overexpression efficiency of Snord3a was evaluated by qRT-PCR analysis in TCMK1 cells (n=3 for each group). (B) Cell viability was measured by CCK8 assay (n=3 for each group). (C) GSH levels and (D) MDA levels in different groups (n=3 for each group). (E-F) Intracellular ROS and lipid peroxidation were detected by flow cytometry using a DHR123 probe and a BODIPY 581/591C11 probe (n=3 for each group). Data were presented as means ± SD. One-way ANOVA test is used for (A, B, C, D, E, and F). **P* < 0.05, ***P* < 0.01, ****P* < 0.001.

**Fig. S8. Snord3a deficiency ameliorates ferroptosis in IRI-induced AKI mice model.** *Snord3a^fl/fl^* and *Snord3a^fl/fl^-GGT1^Cre^* mice were subjected to IRI or sham surgery and harvested 24h later. (A-D) Immunoblotting analysis and quantification of ACSL4, COX2, and GPX4 expression (per group n=6). (E-G) GSH levels, MDA levels, and Iron levels in different groups (per group n=6). (H) Representative mitochondrial morphology was visualized by transmission electron microscope (6500X, scale bar = 1 μm; 17500×, scale bar = 500 nm). Arrow: diminished or vanished mitochondria crista, or ruptured mitochondrial outer membrane. Data were presented as means ± SD. One-way ANOVA test is used for (B, C, D, E, F, and G). **P* < 0.05, ***P* < 0.01, ****P* < 0.001, *****P* < 0.0001.

**Fig. S9. Snord3a meditates ferroptosis through an FBL-independent pathway.** (A) RNA pull-down assay and immunoblotting analysis for the interactions of Snord3a with FBL in TCMK1 cells. (B-D) TCMK1 cells were transfected with Snord3a plasmid or (and) FBL siRNA before 24 h of cisplatin treatment (20 μM, 24h). (B) Cell viability was measured by CCK8 assay (n=3 for each group). (C) GSH levels and (D) MDA levels in different groups (n=3 for each group). Data were presented as means ± SD. One-way ANOVA test is used for (B, C, and D). **P* < 0.05, ***P* < 0.01, ****P* < 0.001, *****P* < 0.0001.

**Fig. S10. CHIRP-Seq of Snord3a.** (A) CHIRP assay and RT-qPCR analysis of the enrichment degree of Snord3a in different probes (n=3 for each group). (B) Relative binding intensity of Snord3a at the transcription start site (TSS) vs input sample. (C) Predicted Snord3a motifs by MEME and transcription factor motifs similar to the predicted Snord3a binding motifs by Tomtom. Data were presented as means ± SD. One-way ANOVA test is used for (A). **P* < 0.05, *****P* < 0.0001.

**Fig. S11. Transfection efficiency of STING in TCMK1 cells.** TCMK1 cells were transfected with negative control siRNA (si-NC) or STING siRNA (si-Sting), or transfected with empty vector plasmid (Vector) or STING overexpression plasmid (Sting). (A) The knockdown efficiency of STING was evaluated by qRT-PCR and immunoblotting analysis (n=3 for each group). (B) The overexpression efficiency of STING was evaluated by qRT-PCR (n=3 for vector plasmid group and n=4 for STING plasmid group) and immunoblotting analysis (n=3 for each group). Data were presented as means ± SD. Two-sided Student’s t test is used for (A, B). **P* < 0.05, ***P* < 0.01, ****P* < 0.001.

**Fig. S12. STING overexpression rescues ferroptosis improvement due to Snord3a silencing in cisplatin-induced TCMK1 cells.** TCMK1 cells were transfected with Snord3a ASO or (and) STING plasmid before 24h of cisplatin treatment (20 μM, 24h). (A-B) Intracellular ROS and lipid peroxidation were detected by flow cytometry using a DHR123 probe and a BODIPY 581/591C11 probe (n=3 for each group). (C) MDA levels and (D) GSH levels in different groups (n=3 for each group). (E) Cell viability was measured by CCK8 assay (n=3 for each group). (F-G) Cell death was detected by flow cytometry using annexin V and 7-AAD sensors (n=3 for each group). Data were presented as means ± SD. One-way ANOVA test is used for (A, B, C, D, E, and F). **P* < 0.05, ***P* < 0.01, ****P* < 0.001, *****P* < 0.0001.

**Fig. S13. Snord3a ASO treatment ameliorates kidney injury in cisplatin-induced AKI mice model.** Mice were injected with the Snord3a ASO or negative control ASO in the mouse kidneys every two days for three times, and a cisplatin-induced AKI mice model was performed after the ASO injection. (A-B) Snord3a expression was evaluated by FISH and RT-qPCR analysis. Representative FISH images of the kidney expressing Snord3a (red) and co-stained with DAPI (blue). Scale bars, 20 μm. n=3 for each FISH group and n=5 for each RT-qPCR group. (C-D) Immunoblotting analysis and quantification of KIM1 and NGAL in the kidney (n=5 for each group). (E) RT-qPCR analysis of *Kim1* and *Lcn2* mRNA in the kidney (n=5 for each group). Data were presented as means ± SD. One-way ANOVA test is used for (A, B, D, and E). ***P* < 0.01, ****P* < 0.001, *****P* < 0.0001.

**Fig. S14. The therapeutical application of Snord3a in IRI-induced AKI mice model.** (A) Schematic diagram of Snord3a ASO treatment in IRI-induced AKI mice model. Briefly, each group was injected with the Snord3a ASO or negative control ASO in the mouse kidneys every two days for three times, and an IRI-induced AKI mice model was performed after the ASO injection. (B) Kidney function was assessed by serum creatinine and BUN levels (per group n=4). (C-D) Representative images of HE and PAS staining of renal sections, and quantification of tubular injury score (per group n=4). Scale bars, 100 μm. (E-H) Representative immunofluorescence images of KIM1 (green) and co-stained with LTL (red), or NGAL (green), and quantification of positive areas (per group n=4). Scale bars, 100 μm. (I) Representative mitochondrial morphology was visualized by transmission electron microscope (6500X, scale bar = 1 μm; 17500X, scale bar = 500 nm). Arrow: diminished or vanished mitochondria crista, or ruptured mitochondrial outer membrane. (J-L) Iron levels, GSH levels, and MDA levels of the kidney (per group n=4). Data were presented as means ± SD. One-way ANOVA test is used for (B, D, F, G, H, J, K, and L). **P* < 0.05, ***P* < 0.01, ****P* < 0.001, *****P* < 0.0001.

**Fig. S15. Snord3a ASO treatment ameliorates kidney injury in IRI-induced AKI mice model.** Mice were injected with the Snord3a ASO or negative control ASO in the mouse kidneys every two days for three times, and an IRI-induced AKI mice model was performed after the ASO injection. (A-B) Snord3a expression was evaluated by FISH and RT-qPCR analysis. Representative FISH images of the kidney expressing Snord3a (red) and co-stained with DAPI (blue). Scale bars, 20 μm. n=3 for each FISH group and n=4 for each RT-qPCR group. (C-E) Immunoblotting analysis and quantification of KIM1 and NGAL in the kidney (n=4 for each group). (F-G) RT-qPCR analysis of *Kim1* and *Lcn2* mRNA in the kidney (n=4 for each group). Data were presented as means ± SD. One-way ANOVA test is used for (A, B, D, E, F, and G). **P* < 0.05, ***P* < 0.01, ****P* < 0.001, *****P* < 0.0001.

**Figure. S16**

**Fig. S16. Representative images of HE and PAS staining of renal tissue in different models.** (A) The cisplatin-induced AKI model in *Snord3a^fl/fl^* and *Snord3a^fl/fl^-GGT1^Cre^* mice. (B) The cisplatin-induced AKI model with c-di-AMP or saline in *Snord3a^fl/fl^* and *Snord3a^fl/fl^-GGT1^Cre^* mice. (C) Snord3a ASO or NC ASO treatment in the cisplatin-induced AKI mice model.

**Supplementary Tables**

**Table S1. Differentially expressed snoRNAs in renal tubular epithelial cells of AKI.**

| **Gene ID** | **Gene name** | **log2FC (cisplatin/control)** | ***P* value** | **Regulate** |
| --- | --- | --- | --- | --- |
| MSTRG.14729 | *Snora17* | 8.270837073 | 1.29184E-15 | Up |
| MSTRG.1232 | *Snord70* | 4.02772497 | 1.50331E-34 | Up |
| MSTRG.23210 | *Snord23* | 3.710532349 | 3.56956E-10 | Up |
| MSTRG.15012 | *Snord90* | 3.222770953 | 1.48847E-54 | Up |
| MSTRG.15867 | *Snord110* | 2.884519853 | 3.36327E-32 | Up |
| MSTRG.19066 | *Snora73b* | 2.596278251 | 6.30241E-07 | Up |
| MSTRG.11598 | *Snora78* | 2.367390885 | 3.63645E-11 | Up |
| MSTRG.1501 | *Snord82* | 2.307373808 | 0.001875477 | Up |
| MSTRG.2893 | *Snord3a* | 2.234144 | 0.010589158 | Up |

**Table S2. Differentially expressed snoRNAs in both cisplatin-treated TCMK1 and HK2 cells.**

| **Gene name** | **log2FC (cisplatin/control)**  **in TCMK1 cells** | **log2FC (cisplatin/control)**  **in HK2 cells** |
| --- | --- | --- |
| *Snora3a* | 8.74396 | 6.13245 |
| *Snord17* | 2.18905 | 2.48171 |
| *Snord23* | 3.97304 | 1.92926 |
| *Snord70* | 4.09927 | 4.91613 |
| *Snord73b* | 0.90296 | 0.97840 |
| *Snora78* | 3.65196 | 3.69508 |
| *Snora82* | 2.30189 | 1.15741 |
| *Snord90* | 2.47433 | 0.49063 |
| *Snord110* | 2.88140 | 0.49063 |

**Table S3. The clinical characteristics of patients with AKI and healthy donors.**

|  | **CON (n=30)** | **AKI (n=30)** | ***P* value** |
| --- | --- | --- | --- |
| Age (years) | 55.00(40.50,62.50) | 57.00(50.25,62.50) | 0.159 |
| Female, n (%) | 11(36.67) | 16(53.33) | 0.127 |
| Albumin (g/L) | 38.15±0.84 | 36.13±1.09 | 0.145 |
| Serum creatinine (µmol/L) | 49.25(40.05,66.00) | 222.00(161.00,427.50) | <0.001 |
| Serum BUN (mmol/L) | 4.85(3.16,9.61) | 11.09(8.41,17.33) | <0.001 |
| eGFR (ml/min/1.73m^2^) | 120.64(103.45,145.92) | 22.49(9.82,33.35) | <0.001 |

**Table S4. The sequence of ASO, SiRNAs, FISH probes, CHIRP probes, and RNA pull down probes.**

| **Name** | **Sequence** |
| --- | --- |
| Snord3a ASO (mouse) | GATCGTTCTCAACTCTCTTG |
| **CHIRP probes** | **Sequence** |
| Odd-1 | 5’GATCCCTGAAAGTATAGTCT 3’ |
| Odd-2 | 5’CCACTCAGACTGTGTCCTCT 3’ |
| Even-1 | 5’CTCAGGAAAGGACACTCTGT 3’ |
| LacZ | 5’TCACGACGTTGTAAAACGAC 3’ |
| **RNA pull down probes** | **Sequence** |
| 3A_Oligo1 (Sense) | 5’AGACUAUACUUUCAGGGAUC3’ |
| 3A_Oligo2 (Sense) | 5’UCAGGGAUCAUUUCUAUAGU3’ |
| 3A_Oligo3 (Sense) | 5’ UUACUAGAGAAGUUUCUCUG3’ |
| 3A_Oligo4 (Sense) | 5’ CCUGAGCGUGAAGCCGGCUU3’ |
| 3A_Oligo5 (Sense) | 5’GCCAUUGAUGAUCGUUCUUC3’ |
| 3A_OligoA1 (Anti-sense) | 5’UAGAAAUGAUCCCUGAAAGU3’ |
| 3A_OligoA2 (Anti-sense) | 5’GAAGAACGAUCAUCAAUGGC3’ |
| 3A_OligoA3 (Anti-sense) | 5’CAGAGAAACUUCUCUAGUAA3’ |

**Table S5. The information of Antibodies**

| **Antibody** | **Cat. Number** | **Company** |
| --- | --- | --- |
| β-actin | 4970 | CST |
| GAPDH | 60004-1-Ig | Proteintech |
| KIM1 | AF1817 | R&D Systems |
| NGAL | AF1857 | R&D Systems |
| COX2 | ab15191 | Abcam |
| ACSL4 | 22401-1-AP | Proteintech |
| NCOA4 | ab86707 | Abcam |
| GPX4 | ab125066 | Abcam |
| RIPK3 | 17563-1-AP | Proteintech |
| RIPK1 | 3493 | CST |
| BAX | ab32503 | Abcam |
| BCL2 | 3498 | CST |
| GSDMD | PD00-18 | HuaBio |
| Caspase1 | SU40-07 | HuaBio |
| Cleaved-Caspase1 | AF4005 | Affinity |
| STING | 50494 | CST |
| Phospho-STING | 19781 | CST |
| P65 | 8242 | CST |
| Phospho-P65 | 3033 | CST |
| TBK | 28397-1-AP | Proteintech |
| Phospho-TBK | D2S9R | CST |
| PKNOX1 | A15301 | ABclonal |
| RAPGEF3 | A4149 | ABclonal |
| BPNT1 | 16461-1-AP | Proteintech |
| PTBP1 | 12582-1-AP | Proteintech |

**Table S6. Sequences of primers for quantitative RT-PCR**

| **Gene name** | **Species** | **Forward primers** | **Reverse primers** |
| --- | --- | --- | --- |
| *Snora17* | Mouse | CCTTCTTCCCAGTCTCGGTTC | GTAGCCTGCCAGCACACAAG |
| *Snord70* | Mouse | CTTTTGGAACTGAATCTAAGTGATT | TCTCAGTGGTAGTGACGAATTT |
| *Snord23* | Mouse | CCATCCTTGCTCCCCGTG | CCCTCAGTCCCCAGGCAA |
| *Snord90* | Mouse | TAGGGCAGATTCTGAGGTGA | AGTTTTCAAGTGTCTTCAGATTTCA |
| *Snord110* | Mouse | TGACTTATATATCTGTCAATCCCCTG | TTGCTCAGACACATGGAGTCGT |
| *Snora73b* | Mouse | ATGAGTGATGACAAGATGTTTCGG | GAATGTCAGCAATAAATGTTACGGTA |
| *Snora78* | Mouse | GGCTTACGCAGTTGATCCAGTA | GAATCTCACCCCCGGCTAC |
| *Snord82* | Mouse | GTGATGAGTGACAAAGGGACT | TTTCATTTCAACCCCATGGTTC |
| *Snord3a* | Mouse | GTGTCCTTTCCTGAGCGTGA | ACTGTGTCCTCTCCCTCTCA |
| *β-actin* | Mouse | CATTGCTGACAGGATGCAGAA | ATGGTGCTAGGAGCCAGAGC |
| *Kim1* | Mouse | CAGGGAAGCCGCAGAAAA | GAGACACGGAAGGCAACCAC |
| *Lcn2* | Mouse | CCAGTTCGCCATGGTATTTT | CCTTGAGGCCCAGAGACT |
| *Sting* | Mouse | CTACATTGGGTACTTGCGGTT | GCACCACTGAGCATGTTGTTATG |
| *β-ACTIN* | Human | CTCCATCCTGGCGCTGT | GCTGTCACCTTCACCGTTCC |
| *SNORD3A* | Human | CGGTGACGGCTCTTGGGTTT | CGGGAAACGGCGACAAAA |
